# Solution growth of NiO nanosheets supported on Ni foam as high-performance electrodes for supercapacitors

**DOI:** 10.1186/1556-276X-9-424

**Published:** 2014-08-22

**Authors:** Hailong Yan, Deyang Zhang, Jinyou Xu, Yang Lu, Yunxin Liu, Kangwen Qiu, Yihe Zhang, Yongsong Luo

**Affiliations:** 1 School of Physics and Electronic Engineering, Xinyang Normal University, Xinyang 464000, People's Republic of China; 2 Key Laboratory of Advanced Micro/Nano Functional Materials, Xinyang Normal University, Xinyang 464000, People's Republic of China; 3 Department of Physics and Electronic Science, Hunan University of Science and Technology, Xiangtan 411201, People's Republic of China; 4 School of Materials Science and Technology, China University of Geosciences, Beijing 100083, People's Republic of China

**Keywords:** NiO nanosheets, Hydrothermal approach, Long cycle life, Flexible supercapacitors

## Abstract

Well-aligned nickel oxide (NiO) nanosheets with the thickness of a few nanometers supported on a flexible substrate (Ni foam) have been fabricated by a hydrothermal approach together with a post-annealing treatment. The three-dimensional NiO nanosheets were further used as electrode materials to fabricate supercapacitors, with high specific capacitance of 943.5, 791.2, 613.5, 480, and 457.5 F g^-1^ at current densities of 5, 10, 15, 20, and 25 A g^-1^, respectively. The NiO nanosheets combined well with the substrate. When the electrode material was bended, it can still retain 91.1% of the initial capacitance after 1,200 charging/discharging cycles. Compared with Co_3_O_4_ and NiO nanostructures, the specific capacitance of NiO nanosheets is much better. These characteristics suggest that NiO nanosheet electrodes are promising for energy storage application with high power demands.

## Background

Supercapacitors, also called electrochemical capacitors, are the most promising energy storage and power output technologies for digital communication devices, hybrid electric vehicles, and other high-power energy sources, which are attributed to the advantages of high power density, short charging time, high cycle efficiency, and long cycle life [1-6]. However, due to the low energy density of current supercapacitor products, nowadays, developing novel electrode materials with enhanced energy density, while maintaining a high power density, good specific capacitance, and cycling stability for supercapacitors, has become a primary research focus. Unfortunately, the practical applications of supercapacitors are largely hindered due to the lack of high-performance electrode materials at a reasonable cost [7-9]. Carbon-based materials and many transition metal oxides have been widely investigated as electrode materials for supercapacitors with notable improvements achieved [10-13]. However, the relatively low specific and volumetric capacitances of carbon-based materials and extremely high cost of the state-of-art RuO_2_ materials have seriously limited their practical application in supercapacitors. The development of nanomaterials, especially metal oxides, will undoubtedly provide a promising solution to enhance the capacitive performance because of their high surface area, and ion transport pathways. Several promising materials, including nickel oxide, cobalt oxide, and manganese oxide, have been intensively studied as advanced electrode materials for supercapacitors [14-16]. Nickel oxide (NiO) has been intensively studied as supercapacitors for its high theoretical specific capacitance of 2,584 F g^-1^[17,18]. In other words, lightweight and flexibility have become one of the most important development trends of portable electronics in these years [19,20]. Whether flexible portable electronics become popular depends on the improvements of the technology, especially by developing the flexible high-performance energy storage devices.

A facile solution method is developed to grow NiO nanonsheets directly on Ni foam, which possess an enhanced electrochemical performance for supercapacitors. Our optimized supercapacitor shows a specific capacitance of 943.5 F g^-1^ at a current density of 5 A g^-1^. It is found that the specific capacitance of NiO nanonsheets is higher than those of Co_3_O_4_ and NiO nanostructures fabricated by the same method. It can be concluded that vertical NiO nanosheets would be particularly suited to the high-performance electrodes for supercapacitors.

## Methods

### Synthesis of NiO nanosheets on Ni foam

**Figure 1:**
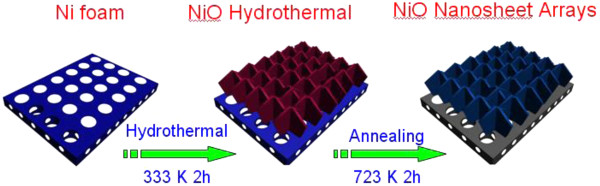
Schematic illustration of the formation processes of the NiO nanosheets.

The NiO nanosheets were synthesized by a simple hydrothermal method (as shown in Figure 1): First, the Ni foam substrate was immersed in a 3 M HCl solution to remove the possible oxide layer on the surface. Then it was cleaned with deionized water and alcohol and dried in air. After the acid etching treatment process, a flat and clean surface is achieved. Second, 0.56 g of NiNO_3_ · 6H_2_O (99.9%, Sigma-Aldrich, St. Louis, MO, USA) was dissolved in a 40-ml mixture containing 3 ml of glycerol and 37 ml of deionized water. After stirring for about 60 min, a transparent solution was obtained and then a certain amount of urea, 0.4 g, was added into the above solution. Third, the obtained solution and the cleaned conductive substrate were transferred into a 50-ml stainless steel autoclave, followed by heating at 200°C for 24 h in an electric oven. After reaction for 24 h, the product was taken out from the solution and cooled down to room temperature. Then the product was cleaned by ultrasonication to remove the loosely attached products on the surface. At last, the substrate was dried at 60°C for further characterization. In order to get crystallized NiO nanosheets, the as-grown precursor nanosheets were annealed in vacuum at a temperature of 450°C for 40 min with a heating rate of 3°C min^-1^. After that, Co_3_O_4_ nanoneedle arrays and NiO powders were fabricated at the same reaction condition for comparison. NiNO_3_ · 6H_2_O and CoNO_3_ · 6H_2_O (99.9%, Sigma-Aldrich) were used as source materials.

### Characterization

The morphology of the synthesized product was examined using field emission scanning electron microscopy (S4800, Hitachi, Chiyoda-ku, Japan). The chemical composition of the product was characterized by X-ray diffraction (XRD; D8 Advance X-ray Diffractometer, Cu K_α_, *λ* = 1.5406 Å, Bruker, Saarbrucken Germany). Raman spectra were recorded on an INVIA Raman microprobe (Renishaw Instruments, Wotton-under-Edge, England) with a 532-nm laser excitation. The thermogravimetric analysis (TGA) curve was performed using a SDT Q600 TA with 100 ml min^-1^ of air flow from 20°C to 600°C at a heating rate of 10°C min^-1^.

The capacitive performance of the samples was tested on a CHI 660E electrochemical workstation (CH Instruments, Chenhua, Shanghai, YP, China) with cyclic voltammetry and chronopotentiometry functions using a three-electrode cell where Pt foil serves as the counter electrode and a saturated calomel electrode (SCE) as the reference electrode. The mass loading of unit area on the Ni foam can be calculated to be 1.78 mg cm^-2^. The electrolyte used was a 3 M KOH aqueous solution.

## Results and discussion

**Figure 2:**
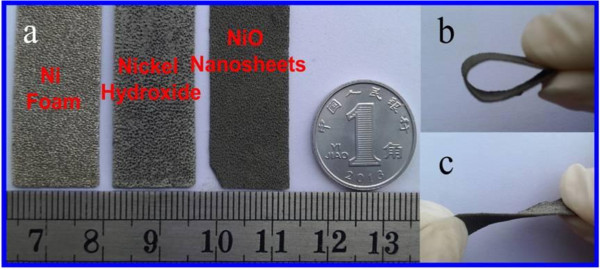
**Optical images. (a)** From left to right: optical photos of the Ni foam, as-grown nickel hydroxide, and NiO nanosheets. **(b, c)** Optical images for the flexible electrode material.

**Figure 3:**
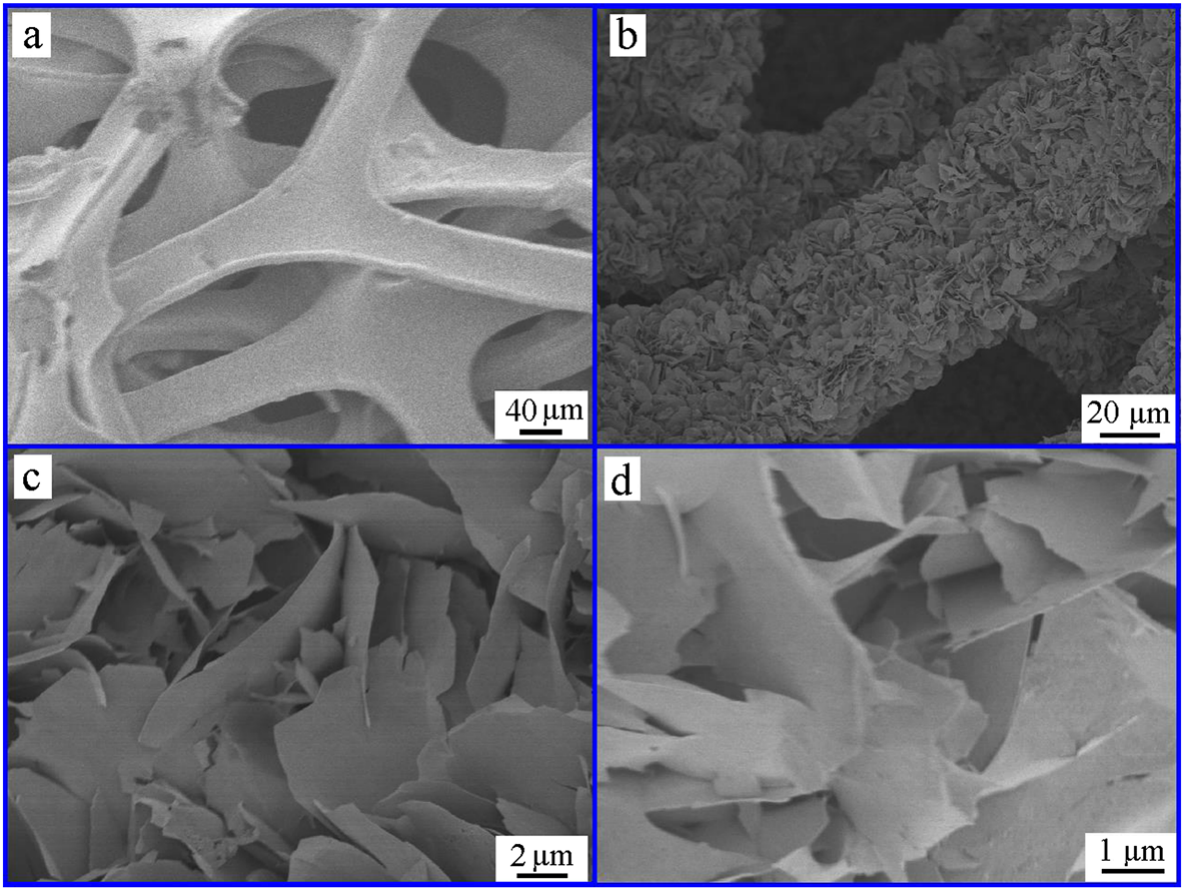
**FESEM images of Ni foam and NiO nanosheets. (a)** Low-magnification FESEM image of the nickel foam. **(b, c)** Low- and high-magnification FESEM images of the NiO nanosheets. **(d)** FESEM images of the NiO nanosheets after charging/discharging for 3,000 cycles.

The optical images of the Ni foam and the annealed NiO nanosheets are shown in Figure 2: from left to right: Ni foam, as-grown nickel hydroxide, and NiO nanosheets (Figure 2a). Figure 2b,c shows the optical images for the flexible electrode material, which can withstand strain relaxation and mechanical deformation. Figure 3a shows a low-magnification field emission scanning electron microscopy (FESEM) image of the nickel foam substrate. Figure 3b is the FESEM image of the NiO nanosheets supported on the nickel foam substrate. As can be seen, the nanosheets are uniformly grown on Ni foam. To further reveal its microstructure, Figure 3c shows a high-magnification FESEM image of the NiO nanosheet with a lateral size of several hundred nanometers and a thickness of several nanometers. This characteristic will benefit electron transmission. Figure 3d shows the FESEM image of the NiO nanosheets after charging/discharging for 3,000 cycles at a current density of 11.8 mA cm^-2^. As can be seen, the morphology of the nanosheets is retained well.

**Figure 4:**
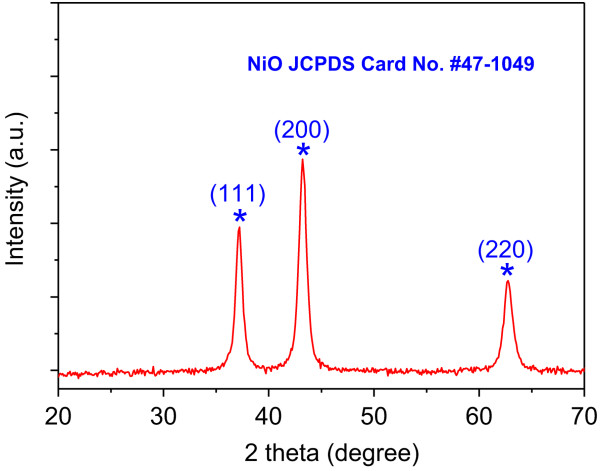
XRD pattern of NiO nanosheets.

The NiO nanosheets are further characterized by using X-ray diffraction and Raman spectroscopy. Typical XRD patterns of the annealed NiO nanosheets are shown in Figure 4. All of the reflections in the XRD pattern can be indexed to face-centered cubic phase NiO (JCPDS card no. #47-1049). The three characteristic peaks at 37.2°, 43.2°, and 62.8° correspond to the (111), (200), and (220) diffraction planes, respectively. The high peak intensity indicates that the NiO nanosheets are of high crystallinity. No peaks from other phases are detected, indicating that the product is of high purity. Moreover, no peaks from the Ni substrate are detected, suggesting that the NiO nanosheets are uniformly grown upon the Ni foam surface. The average crystallite size calculated using the Scherrer equation based on the half-width of the (200) peak is about 14.3 nm. Additional file 1: Figure S1 shows the Raman spectrum of NiO. Three Raman peaks located at about 436, 519, and 1,154 cm^-1^ are observed in the spectra, corresponding to the shaking peaks of NiO. The former two peaks could be attributed to the first-order transverse optical (1TO) vibration mode and longitudinal optical (1LO) phonon modes of NiO, respectively. The peaks at 1,154 cm^-1^ could be assigned to two-phonon (2P) 2LO modes of NiO. Such a result further confirms that the crystalline structure of the NiO nanostructure has been obtained.

**Figure 5:**
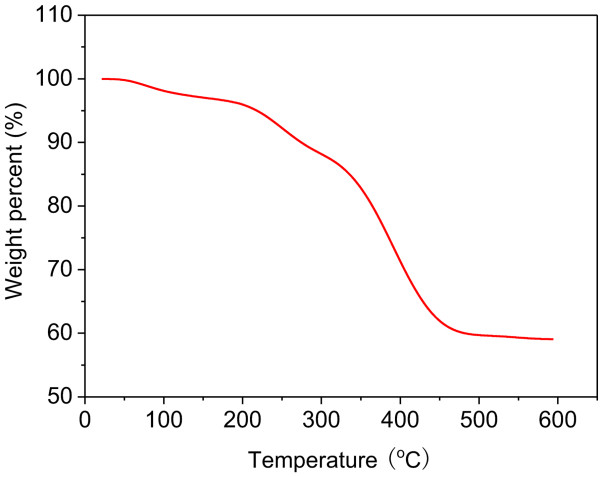
**TGA curve of Ni(OH)**_**2 **_**precursor from 20°C to 600°C.**

The TGA curve of the Ni(OH)_2_ nanosheets is shown in Figure 5. The initial weight loss of about 4% between 20°C and 200°C is due to the removal of physically adsorbed water molecules. The rapid weight loss of about 34% between 200°C and 450°C is due to the removal of the crystalline water molecules and the decomposition of Ni(OH)_2_. Beyond 500°C, all the intercalated water molecules and NiO are formed as the final product. Moreover, there is no obvious weight loss when the temperature is higher than 500°C, and hence, it can be concluded that all Ni(OH)_2_ was converted into NiO.

**Figure 6:**
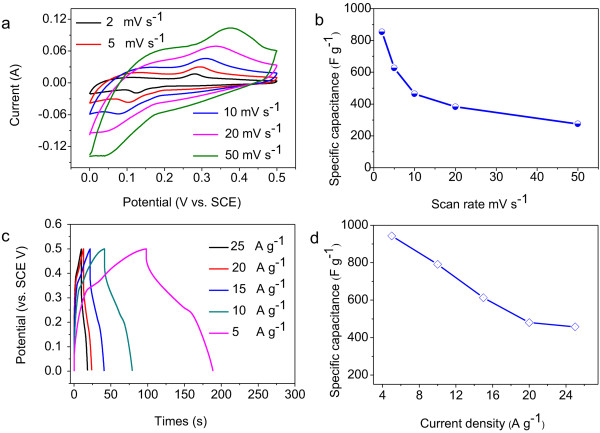
**Electrochemical characterizations of the NiO nanosheets on Ni foam as electrodes for supercapacitors. (a)** CV curves at various scan rates ranging from 2 to 50 mV s^-1^. **(b)** Average specific capacitance at various scan rates. **(c)** Charging/discharging voltage curves at various current densities ranging from 5 to 25 A g^-1^. **(d)** Specific capacitance of NiO nanosheets at various discharge current densities.

To highlight the merits of the unique architecture, we directly apply the hybrid structure of ultrathin NiO nanosheets as an electrode for supercapacitors. Figure 6a shows the cyclic voltammetry (CV) curves of the NiO nanosheet electrode with various sweep rates ranging from 2 to 50 mV s^-1^. A distinct pair of current peaks can be identified during the cathodic and anodic sweeps, whose intensity increases with the scan rate. It can be attributed to the following reversible redox reaction:

(1) $$\text{NiO}+{\text{OH}}^{-}↔\text{NiOOH}+{\text{e}}^{-}$$

suggesting the pseudocapacitive characteristic of the NiO nanosheets. A pair of redox peaks is located at around 0.125 and 0.281 V with the scan rate of 2 mV s^-1^. With the 25-fold increase in the sweep rate from 2 to 50 mV s^-1^, the position of the cathodic peak shifts slightly from 0.125 to 0.047 V. This observation suggests a relatively low resistance of the electrode because of the good contact between the electroactive NiO nanosheets and the conductive Ni foam substrate. The specific capacitance of the electrode can be calculated from the CV curves according to the following equation [21]:

(2) $$C=\int IdV/\left(\mathrm{\upsilon mV}\right)$$

where *C* is the specific capacitance (F g^-1^) based on the mass of the electroactive materials, *I* is the response current density (A), *V* is the potential (V), *v* is the potential scan rate (mV s^-1^), and *m* is the mass of the electroactive materials in the electrodes (g). Based on these CV curves, the specific capacitance of the sample can be calculated to be 817.6, 602.3, 438.4, 367.6, and 254.3 F g^-1^ at the scan rates of 2, 5, 10, 20, and 50 mV s^-1^, respectively (Figure 6b). The charging/discharging measurements are carried out in a 3 M KOH electrolyte at various current densities ranging from 5 to 25 A g^-1^, as shown in Figure 6c. The specific capacitance of the electrode can be calculated from the CV curves according to the following equation [22]:

(3) $$C_{m}=I\Delta t/\left(\Delta \text{Vm}\right)$$

where *C*_*m*_ (F g^-1^) is the specific capacitance, *I* (A) is the discharge current, Δ*t* (s) is the charging/discharging time, Δ*V* (V) is the voltage window for discharge, and *m* (g) is the mass of the active NiO material in the electrode. Thus, the specific capacitance can be calculated to be 943.5, 791.2, 613.5, 480, and 457.5 F g^-1^ at the scan rates of 5, 10, 15, 20, and 25 A g^-1^, respectively (Figure 6d). The specific capacitance of NiO nanosheets is much higher than that of NiO nanobelts, nanorods, and nanosheets reported previously [22-26]. To evaluate the important role of NiO nanosheets for high-performance electrodes, the specific capacitances of Co_3_O_4_ nanoneedles and NiO powders are also tested at the scan rates of 5, 10, 15, 20, and 25 A g^-1^, respectively. The specific capacitances of NiO nanosheets win out over those of Co_3_O_4_ nanoneedles and NiO powders (Additional file 1: Figure S2).

**Figure 7:**
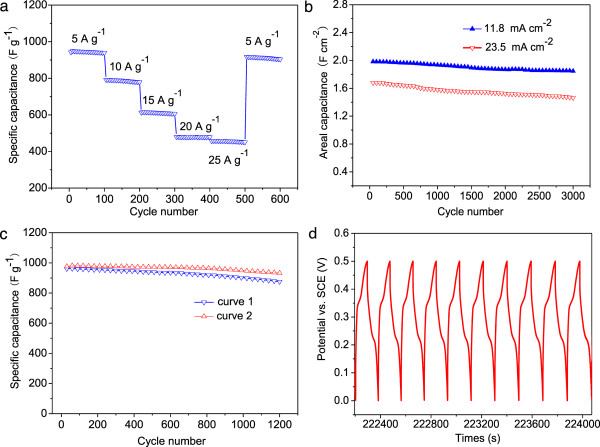
**Cycling performance, capacitance, specific capacitance vs. charging/discharing cycle number, and galvanostatic charge and discharge voltage curves. (a)** Cycling performance of different samples at progressively varying current densities. **(b)** The capacitance as a function of cycle number at constant current densities of 11.8 and 23.5 mA cm^-2^. **(c)** The specific capacitance vs. charging/discharging cycle number of bent and flat electrodes at a current density of 5 A g^-1^. **(d)** Galvanostatic charge and discharge voltage curves of the flat electrode at a current density of 5 A g^-1^ for the last 10 cycles.

Different rates of charging/discharging are used to investigate the high rate capability of the NiO nanosheet electrode as shown in Figure 7a. The Ni foam-supported NiO nanosheet electrode is first cycled with a current density of 5 A g^-1^, and then the current density is increased to 10, 15, 20, and 25 A g^-1^, successively. Along with the increment of current density, the corresponding specific capacitances within 100 cycles are measured at 942.6, 791.4, 613.2, 479.6, and 457.1 F g^-1^, respectively. As can be seen in Figure 7a, when the current density is decreased to 5 A g^-1^ again, a specific capacitance is recovered at 904.2 F g^-1^, about 95.9% of the specific capacitance of the initial 100 cycles at 5 A g^-1^, illustrating an excellent rate capability. In addition, the cycling stability of the NiO nanosheet electrode is also evaluated by the repeated charging/discharging measurement at constant current densities of 11.8 and 23.5 mA cm^-2^, as shown in Figure 7b. When a discharge current density of 11.8 mA cm^-2^ is applied, the areal capacitance reaches a value of 1.98 F cm^-2^ in the 50th cycle. After 3,000 cycles, the supercapacitor displays an excellent long cycle life with only 6.8% deterioration of its initial specific capacitance, demonstrating superior long-term electrochemical stability [27]. Even at a high charging/discharging current density of 23.5 mA cm^-2^, the areal capacitance can still reach 1.68 F cm^-2^ in the first cycle and gradually decreases to 1.46 F cm^-2^ over 3,000 cycles, with a capacitance loss of 13.1%. Figure 7c (curve 1) displays the specific capacitance vs. charging/discharging cycle number of bent electrode at a current density of 5 A g^-1^. At a discharge current density of 5 A g^-1^, the areal capacitance can still reach 961.4 F g^-1^ in the first cycle and gradually decreases to 875.6 F g^-1^ over 1,200 cycles, equivalent to 91.1% of the capacitance delivered in the first few cycles. Figure 7c (curve 2) displays the specific capacitance of flat electrode at a current density of 5 A g^-1^. Remarkably, the NiO nanosheets exhibit an excellent retention capacitance of 932.6 F g^-1^ at the end of 1,200 charging/discharging cycles [27-29], equivalent to 95.3% of the capacitance delivered in the first few cycles. Figure 7d shows the charging/discharging voltage curves of the NiO nanosheets at a current density of 5 A g^-1^ for the last 10 cycles, and a coulombic efficiency of ≈ 100% can be reached for each cycle.

The improved electrochemical performance could be related to the following structural features. Firstly, the aligned NiO nanosheets with a high surface area facilitate ion diffusion from the electrolyte to each nanosheets, making full use of the active materials [30]. Secondly, the vertical NiO nanosheets could ensure good mechanical adhesion, and more importantly, vertical nanosheets can build up a shortcut and high-speed bridge between the current collector and active materials (Additional file 1: Figure S3). Thirdly, Ni foam as the platform for sustaining nanosheets can withstand strain relaxation and mechanical deformation, preventing the electrode materials from seriously swelling and shrinking during the insertion-deinsertion process.

## Conclusions

Well-aligned NiO nanosheets are fabricated by a hydrothermal approach, and it is used as binder-free electrodes for supercapacitors. The ultrathin NiO nanosheets supported on the nickel foam is able to deliver areal capacitances of 1.98 and 1.68 F cm^-2^ at current densities of 11.8 and 23.5 mA cm^-2^, respectively. The vertical NiO nanosheets on the substrate can withstand strain relaxation and mechanical deformation. When the electrode material is bent, it can still retain 91.1% of the initial capacitance after 1,200 charging/discharging cycles. The specific capacitance of NiO nanosheets is much higher than those of Co_3_O_4_ and NiO nanostructures. Such highly integrated binder- and additive-free electrodes made by electroactive NiO nanosheets might hold some potential for the fabrication of high-performance flexible energy storage devices.

## Competing interests

The authors declare that they have no competing interests.

## Authors' contributions

HY carried out the sample preparation, performed all the analyses, and wrote the paper. YL and KQ participated on its analysis. DZ, JX, YXL, YZ, and YSL directed the research and made corrections to the manuscript. All authors read and approved the final manuscript.

## Supplementary Material

Additional file 1 **Supporting information.** Raman spectra of NiO nanosheets (**Figure S1**). Specific capacitance of NiO nanosheets, Co_3_O_4_ nanoneedles, and NiO powders at various discharge current densities (**Figure S2**). Schematic of the electronic transport in NiO nanosheets (**Figure S3**). Click here for file
