# Supplementary material for: Solution growth of NiO nanosheets supported on Ni foam as high-performance electrodes for supercapacitors
Source: Nanoscale Res Lett. 2014 Aug 22;9(1):424. doi: 10.1186/1556-276X-9-424 (PMC4177676; doi:10.1186/1556-276X-9-424)
Supplement: Additional file 1 — Supporting information. Raman spectra of NiO nanosheets (Figure S1). Specific capacitance of NiO nanosheets, Co3O4 nanoneedles, and NiO powders at various discharge current densities (Figure S2). Schematic of the electronic transport in NiO nanosheets (Figure S3). [file 1556-276X-9-424-S1.doc]

**Solution Growth of NiO Nanosheets Supported on Ni Foam as High-Performance Electrodes for Supercapacitors**

Hailong Yan*^1,2^*

Email: yanhailong2005@126.com

Deyang Zhang*^1,2^*

Email: zhangdeyang329@163.com

Yang Lu*^1,2^*

Email: luyang.181@163.com

Yunxin Liu*^3^*

Email: lyunxin@163.com

Kangwen Qiu*^1,2^*

Email: ekwqiu@163.com

Yihe Zhang*^3^*

Email: [zyh@cugb.edu.cn](mailto:zyh@cugb.edu.cn)

Yongsong Luo*^1,2*^*

* Corresponding author

Email: eysluo@163.com

*^1^ School of Physics and Electronic Engineering, Xinyang Normal University, Xinyang 464000, P. R. China*

*^2^ Key Laboratory of Advanced Micro/Nano Functional Materials, Xinyang Normal University, Xinyang 464000, P. R. China*

*^3^ School of Materials Science and Technology, China University of Geosciences, Beijing 100083, P. R. China*

**Figure S1. Room temperature Raman spectrum of NiO in the range of 200-1400 cm^-1^.**

**Figure S2.** Specific capacitance of NiO nanosheets, Co_3_O_4_ nanoneedles, and NiO powders at various discharge current densities.


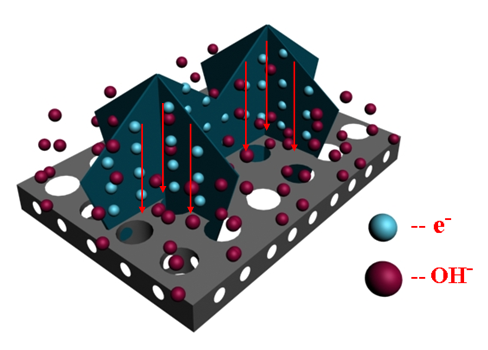


**Figure S3. Schematic of the electronic transport in NiO nanosheets.**
